# Supplementary material for: Effect of ATR Inhibition in RT Response of HPV-Negative and HPV-Positive Head and Neck Cancers
Source: Int J Mol Sci. 2021 Feb 3;22(4):1504. doi: 10.3390/ijms22041504 (PMC7913134; doi:10.3390/ijms22041504)
Supplement: Supplementary file 1 [file ijms-22-01504-s001.pdf]

Supplementary Figure 1

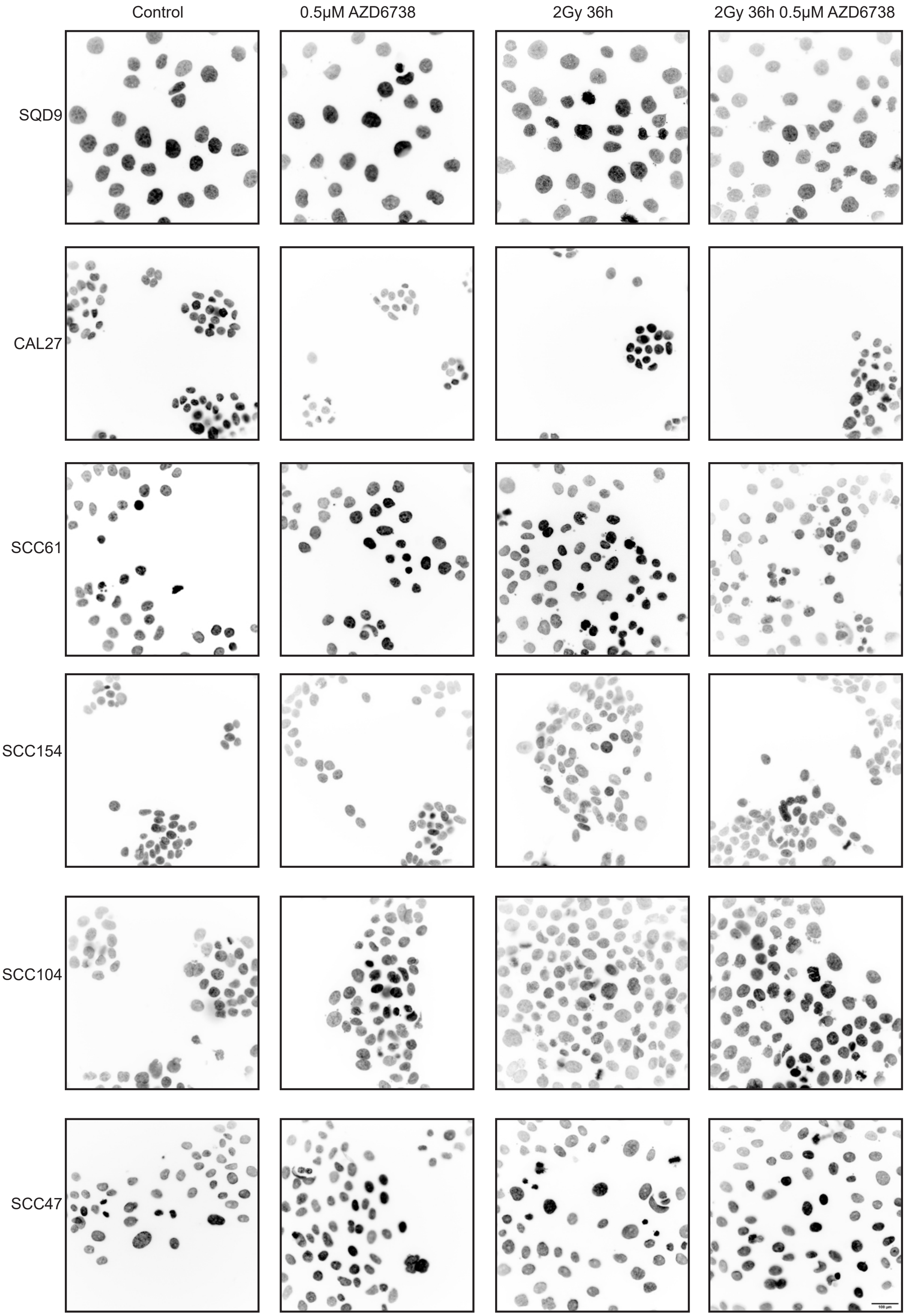

Representative images of the micronuclei assay. HPV-negative (SQD9, CAL27 and SCC61) and HPV-positive (SCC154, SCC104 and SCC47) HNSCC cells were exposed to 0.5µM of AZD6738 and/or 6Gy of RT to assess the percentage of micronucleated cells to the total number of cells. Immunofluorescence images were acquired with In Cell Analyzer 2000 (GE Healthcare, Chicago, IL, USA). Images were inverted in imageJ. scale bar=100µm.
